# Differential neuromuscular training effects onACL injury risk factors in"high-risk" versus "low-risk" athletes

**DOI:** 10.1186/1471-2474-8-39

**Published:** 2007-05-08

**Authors:** Gregory D Myer, Kevin R Ford, Jensen L Brent, Timothy E Hewett

**Affiliations:** 1 Sports Medicine Biodynamics Center and Human Performance Laboratory, Cincinnati Children's Hospital Medical Center, Cincinnati, USA; 2 Graduate Program in Athletic Training, Rocky Mountain University of Health Professions, Provo, USA; 3 Department of Kinesiology and Health Promotion, University of Kentucky, Lexington, USA; 4 Department of Biomedical Engineering and the College of Allied Health Sciences, Department of Rehabilitation Sciences, University of Cincinnati, Cincinnati, USA

## Abstract

**Background:**

Neuromuscular training may reduce risk factors that contribute to ACL injury incidence in female athletes. Multi-component, ACL injury prevention training programs can be time and labor intensive, which may ultimately limit training program utilization or compliance. The purpose of this study was to determine the effect of neuromuscular training on those classified as "high-risk" compared to those classified as "low-risk." The hypothesis was that high-risk athletes would decrease knee abduction moments while low-risk and control athletes would not show measurable changes.

**Methods:**

Eighteen high school female athletes participated in neuromuscular training 3×/week over a 7-week period. Knee kinematics and kinetics were measured during a drop vertical jump (DVJ) test at pre/post training. External knee abduction moments were calculated using inverse dynamics. Logistic regression indicated maximal sensitivity and specificity for prediction of ACL injury risk using external knee abduction (25.25 Nm cutoff) during a DVJ. Based on these data, 12 study subjects (and 4 controls) were grouped into the high-risk (knee abduction moment >25.25 Nm) and 6 subjects (and 7 controls) were grouped into the low-risk (knee abduction <25.25 Nm) categories using mean right and left leg knee abduction moments. A mixed design repeated measures ANOVA was used to determine differences between athletes categorized as high or low-risk.

**Results:**

Athletes classified as high-risk decreased their knee abduction moments by 13% following training (Dominant pre: 39.9 ± 15.8 Nm to 34.6 ± 9.6 Nm; Non-dominant pre: 37.1 ± 9.2 to 32.4 ± 10.7 Nm; p = 0.033 training X risk factor interaction). Athletes grouped into the low-risk category did not change their abduction moments following training (p > 0.05). Control subjects classified as either high or low-risk also did not significantly change from pre to post-testing.

**Conclusion:**

These results indicate that "high-risk" female athletes decreased the magnitude of the previously identified risk factor to ACL injury following neuromuscular training. However, the mean values for the high-risk subjects were not reduced to levels similar to low-risk group following training. Targeting female athletes who demonstrate high-risk knee abduction loads during dynamic tasks may improve efficacy of neuromuscular training. Yet, increased training volume or more specific techniques may be necessary for high-risk athletes to substantially decrease ACL injury risk.

## Background

Female athletes are reportedly 4 to 6 times more likely to sustain a sports-related non-contact ACL injury than male athletes [1-3]. Altered neuromuscular strategies during the execution of sports movements, which manifest in resultant lower limb joint mechanics (motions and loads), may increase the risk of ACL injury in girls and women [4-8]. Knee abduction measures quantified during drop jump tasks, predict ACL injury risk in young female athletes [6]. Such findings have led to the development of specific neuromuscular training interventions designed to reduce risk factors, particularly in females [9-13]. Initial evidence indicates that these programs likely reduce the potential for ACL injury in female athletes [14]. However, recent findings also indicate that even with more widespread education and institution of ACL injury interventions over the recent years, female athletes who participate in the high-risk sports have not decreased their incidence of injury [15]. The disparity between positive laboratory results and actual effects on injury outcomes in high-risk female populations suggests a missing link between current research and clinical applications for neuromuscular training interventions.

One reason for the disparity between laboratory results and incidence outcomes may be related to the difficulty in implementation of the techniques previously found to be successful in altering biomechanics and/or injury risk. Interventions currently utilized in ACL injury prevention programs often involve an entire team, which requires large time commitments and may be perceived by coaches or players to detract from sport specific skill training. Such constraints may deter coaches from instituting ACL injury interventions into their pre-season or in-season conditioning programs [16-18]. However, if pre-season testing methods could identify an athlete who is at high-risk for an ACL injury, coaches may more likely institute and remain committed to injury prevention programs for the identified high-risk athletes.

Currently, there are no published comparisons on the effects of neuromuscular training on biomechanical measures linked to increased ACL injury that distinguish between athletes who are classified as either "high-risk" or "low-risk" for ACL injury. The purpose of this pilot study was to determine the effect of neuromuscular training on those athletes classified as high-risk compared to those classified as low-risk based on their pre-test knee abduction moments. The hypothesis was that high-risk athletes would decrease knee abduction moments during a drop vertical jump after completing a training program, while low-risk and control athletes would not show changes with training.

## Methods

### Subjects

Intervention (n = 18; 16.0 ± 1.0 yrs) and control (n = 11; 16.7 ± 0.9 yrs) subjects from an area high school girls' soccer and basketball teams were recruited to participate in this study. Parents or guardians signed informed consent approved by the Institutional Review Board and assent from the child participants were obtained prior to study participation. Pre-testing occurred one week prior to the first day of training, and post-test measures were recorded approximately 8 weeks after the pretest on all subjects (4 days after the final training session). The outcome measure of knee abduction was quantified during the DVJ.

Logistic regression analysis indicated maximal sensitivity and specificity for prediction of ACL injury risk using external knee abduction (25.25 Nm cutoff) during the DVJ was determined in a previous cohort of female athletes [6]. Based on these data, 12 study subjects (and 4 controls) were grouped into the high-risk (mean right and left knee abduction moment >25.25 Nm) and 6 subjects (and 7 controls) were grouped into the low-risk (knee abduction ≤ 25.25 Nm) categories using mean right and left leg knee abduction moments. The training sessions were conducted by National Strength and Conditioning Association certified professionals and staff members and utilized similar training techniques described previously in the literature [10,11,19,20]. The pre-established compliance criterion required that each subject participate in at least two-thirds (12 of 18) of the training sessions to be included in the study. The height (mean ± SD) of the participants in the intervention group was 165.5 ± 6.5 cm and body mass was 64.6 ± 10.4 kg. In the control group the height and body mass were 168.9 ± 9.1 cm and 64.0 ± 7.9 kg.

### Procedures

Each subject had 37 retroreflective markers placed on: the sacrum, left PSIS, sternum and bilaterally on the shoulder, elbow, wrist, ASIS, greater trochanter, mid thigh, medial and lateral knee, tibial tubercle, mid shank, distal shank, medial and lateral ankle, heel, dorsal surface of the midfoot, lateral foot (5^th ^metarsal) and toe (between 2^nd ^and 3^rd ^metatarsals). A static trial was first collected in which the subject was instructed to stand still and was aligned with the laboratory coordinate system. This static measurement was used as each subject's neutral (zero) alignment with subsequent measures referenced to this position.

Once the static trial was completed, subjects were positioned at the top of a 31 cm box with their feet positioned 35 cm apart (distance measured between toe markers) [4]. The subjects were instructed to perform a drop vertical jump (DVJ) by dropping directly down off the box and immediately performing a maximum vertical jump. The DVJ was employed in order to examine landing mechanics that may be related to the mechanics of similar movements in both soccer and basketball. Also, measures of knee abduction moments recorded during a drop vertical jump reportedly provide good within session and between session reliability over similar time intervals of the current study design (~7 weeks) [21]. The first landing on the force platforms (i.e. the drop from the box) was used for analysis. Three successful trials were recorded for each subject during both pre and post-testing.

Trials were collected with a motion analysis system (EVaRT Version 4, Motion Analysis Corporation) with eight digital cameras (Eagle cameras, Motion Analysis Corporation) positioned in the laboratory and sampled at 240 Hz. The motion analysis system was calibrated with a two-step process prior to data collection. In the first step a static calibration frame was used to orient the cameras with respect to the laboratory coordinate system, and the second step used dynamic wand data to fine tune camera positions, calculate the lens distortion maps, and calculate the lens focal length. Two force platforms (AMTI) were sampled at 1200 Hz and time synchronized with the motion analysis system. The force platforms were embedded into the floor and positioned 8 cm apart so that each foot would contact a different platform during the drop vertical jump.

### Data analyses

Data were imported into Visual 3d (Version 3.65, C-Motion, Inc.) and MATLAB (Version 7.0, The Mathworks) for data reduction and analysis. The vertical ground reaction force (VGRF) data were utilized to calculate initial contact (IC) with the ground immediately after the subject dropped from the box. IC was defined when VGRF first exceeded 10 N. Toe off (TO) was subsequently calculated after IC when the VGRF fell below 10 N. To minimize possible peak impact errors induced by different force and video cutoff frequencies, the force plate data and marker trajectories were filtered through a low-pass fourth order Butterworth filter at the same cutoff frequency of 12 Hz prior to joint moment calculations [22]. These data were used to calculate joint moments using inverse dynamics[23]. Net external moments are described in this paper and represent the external load on the joint.

### Statistical analyses

For each trial, peak (maximum) stance phase values of knee abduction moment were determined. Statistical means and standard deviations for each variable were then calculated for each group. A logistic regression analysis was performed on historical knee abduction moment data to determine a cut-off value that would provide the maximal sensitivity and specificity for prediction of ACL injury risk during a DVJ [6]. A mixed-design repeated measures ANOVA (2 × 2 × 2) was used to test for the main effect and interactions of time (pre vs. post-test), risk factor (high-risk vs. low-risk) and side (dominant vs. non-dominant) on the dependant variable knee abduction moment variable recorded during the DVJ in the training group. A matched mixed-design repeated measures ANOVA was utilized on control subject data. An exploratory alpha level of 0.05 was used to determine statistical significance in all comparisons. Statistical analyses were conducted in SPSS for Windows, Version 12.0 (SPSS Inc., Chicago, Illinois).

## Results

Logistic regression analysis indicated maximal sensitivity and specificity for prediction of ACL injury risk. External knee abduction moment required a cut-off at 25.25 Nm during a drop vertical jump. Based on these data, 12 study subjects (and 4 controls) were grouped into the "high-risk" (mean dominant and non-dominant knee abduction moment >25.25 Nm) and 6 subjects (and 7 controls) were grouped into the "low-risk" (knee abduction ≤ 25.25 Nm) categories using mean pretest values collected prior to training during a DVJ. High-risk and low-risk subjects were not different based on performance measures (P > 0.05). High risk subjects demonstrated a drop vertical jump height of 0.44 ± 0.04 m and low-risk participants leaped 0.44 ± 0.042 m during the DVJ.

The repeated measures ANOVA showed a training X risk factor group interaction. For the athletes classified as "high risk," there was an effect of decreased knee abduction moments with training on both the dominant and non-dominant side. Prior to training, athletes classified as high-risk demonstrated a mean (± 1 SD) knee abduction torque of 39.9 ± 15.8 Nm on their dominant side and 37.1 ± 9.2 Nm on their non-dominant side. Following training, the high-risk athletes reduced their peak knee abduction torque during the DVJ by 13% on both legs. That decrease, on average for both the dominant and non dominant leg, was a reduction of 5 Nm (right side post 34.6 ± 9.6 Nm, left side post: 32.4 ± 10.7 Nm; p= 0.033). There were no other significant interactions or main effects demonstrated by the intervention subjects.

Female athletes grouped into low-risk demonstrated a mean knee abduction torque of 14.8 ± 8.8 Nm on their dominant side and 14.5 ± 6.4 Nm on the non-dominant side. These low-risk participants showed no significant effect of training on knee abduction torque, with post-test knee abduction moments of 17.6 Nm ± 10.2 for the dominant side and 14.7 Nm ± 7.0 on the non-dominant side. In addition, control subjects classified as either high or low-risk did not demonstrate any main effects or interactions between pre- and post-test measures (p > 0.05).

A linear regression analysis was performed to further examine the potential association between the pre-test measures of knee abduction moment and the change in this variable with training. In the trained group, pre-test knee abduction moment predicted 40% of the variance in the reduction of coronal plane knee loads measured after training (SEE 5.99, p = 0.001). In contrast, the control group showed no similar causal relationship of pre-test knee abduction measures to change in post test measure (R = 0.03; SEE 7.73, p = 0.880).

## Discussion

High-risk female athletes may demonstrate a preferential, increased coronal plane load strategy, as opposed to a sagittal plane load absorption strategy, during dynamic sport related activities, which may destabilize the knee and load the ACL [4-7,19,24-27]. This strategy may be an ineffective technique for force dissipation during landing and cutting tasks. Moreover, it may also increase the risk for ACL injury [6]. When neuromuscular training programs are implemented, female athletes achieve improvements in athletic performance, movement biomechanics and reduced ACL injury risk [9-11,17-19,28,29]. Though these data may have been collected before many programs were widely instituted, the known positive effects measured in laboratories have not led to decreased national injury rates [15]. The lack of overall reduction of injuries suggests that further work is needed to determine the most efficient training methods. If determined, these program may be more effective to influence coaches and athletes to institute effective programs and ultimately reduce ACL injury rates. The current study results, which define the potential to target high-risk females based on their knee abduction moments (>25.25 Nm), give evidence for the efficacy of a more "targeted" approach to the current neuromuscular training paradigms.

Cadaveric and modeling studies have shown that increased knee abduction loads directly elicit large ACL loads [30,31]. In vivo data also indicates that females who demonstrate increased knee abduction during landing activities are at increased risk to sustain an ACL injury during competitive play [6]. Increased neuromuscular control of dynamic lower extremity valgus, especially knee abduction, may be necessary to prevent loads that can cause ACL injury[6,7,32,33]. Cumulatively, these data may indicate that interventions aimed specifically at reducing ACL injury risk in female athletes should focus to reduce mechanisms that underlie increased knee abduction loads [6,20].

The current study design focused exclusively on evaluating the training effects on knee abduction load. This was done by utilizing abridged versions of a comprehensive training protocol shown to alter biomechanical factors related to increased ACL injury risk in female athletes [6,11,34]. It was hypothesized that high-risk female athletes would decrease knee abduction moments after training, while low-risk and control athletes would not show changes at post-test measurement. The results of this study supported the hypothesis, indicating that females that demonstrate increased knee abduction loads (>25.25 Nm) may be able to reduce this torque through training, while low-risk athletes may not reap as much benefit from the training program. If this is the case, a logical extension suggests that it is more important to target athletes identified as "high-risk" for injury prevention training programs than those identified as "low-risk." Hewett et al. [19] evaluated the available literature and developed a research design to test the effects of neuromuscular training on measures of lower extremity neuromuscular control. They determined that female athletes can demonstrate either a net knee abduction torque or knee adduction torque when landing from a jump. When evaluating only those females who displayed a predominately net knee abduction torque they determined that significantly reduced peak landing forces and knee abduction torques could be attained after six-weeks of neuromuscular training. While the sample size of females evaluated in that study who demonstrated an overall net knee abduction moment was relatively small (n = 4), the data provides initial evidence that relatively large (> 50%) reductions in coronal plane moments can be achieved with preseason neuromuscular training [19].

Lephart and colleagues [12] evaluated the effects of two different protocols comprised exclusively of plyometrics or basic resistance training (20–30 repetitions of bodyweight and band resistance exercises) on 27 high school female athletes to determine the potential biomechanical effects on female athletes. They found that neither training protocol significantly reduced the knee abduction torque. Myer et al. evaluated a comprehensive neuromuscular training protocol that was developed to reduce ACL injury risk and improve sports related performance measures [11]. This comprehensive protocol successfully reduced knee abduction torques by 21%, but the potentially larger training effects may have been masked by low-risk females, as evidenced by the current study subjects classified as low-risk The low-risk subjects did not change their coronal plane mechanics with similar training regimens. If the subjects in these previous studies had been split into low-risk and high risk categories similar to the current study, larger reductions in knee abduction torque may have been observed, especially in athletes trained with plyometrics who reduced their maximum coronal plane knee torque by 36% (12.5 Nm but was not significant) [12].

To further evaluate the effectiveness of neuromuscular training a systematic review of the published literature was previously performed, which yielded six published interventions targeted towards ACL injury prevention in female athletes[14,16,18,29,35-37]. Four of six significantly reduced knee injury incidence and three of six significantly reduced ACL injury incidence in females. A meta analysis of these six studies demonstrated a significant effect of neuromuscular training programs on ACL injury incidence in female athletes (test for overall effect Z = 4.31, P < 0.0001) [14].

Following the reports of positive biomechanical and epidemiological effects of interventions targeted to reduce ACL injuries in female athletes, the problem was evaluated with recent longitudinal data to determine if the incidence and severity of the problem had changed. Agel et al. [15] performed a thirteen year (1989–2002) retrospective epidemiological study to determine the trends in ACL injury rates of National Collegiate Athletic Association soccer and basketball athletes. These authorsreported that male soccer players demonstrated a significant decrease in ACL injuries, whereas female soccer athletes showed no change over the same time period. Both female basketball and soccer players showed no change in the rates of non-contact ACL injuries over the study period and the magnitude of difference in rates (3.6 X) between their male counterparts remained unchanged[15]. While these retrospective data analyses provide no evidence for decreased rates of ACL injuryover the study period, they may not directly reflect the effect or lack of effect of neuromuscular training programs on actual ACL injury incidence. The data collected and analyzed in the Agel et al. investigation ended in 2002[15]. During this time, the positive benefits of neuromuscular training were just being published in medicaljournals and may not have been adopted into general practice by coaches and athletes. Future prospective, longitudinalinvestigations may show the positiveeffects of neuromuscular training on reduction ofknee injuries in female athletes.

In contrast to the findings of Agel et al.,[15] Gilchrist et al. performed a large scale study to determine the incidence of ACL injury rate in a cohort of the same NCAA collegiate female athletes. This randomized controlled trial (RCT) studied interventional training as part of the practice and game warm-up. The authors demonstrated a significant reduction in non-contact ACL injuries in the study group during one competitive season of soccer [37]. In support of the Agel et al. findings, Pfeiffer and colleagues found a 20 minute plyometric training regimen performed twice a week had no effect on ACL injury high school female athletes[38]. In addition, Grandstrand and colleagues found that a commercially marketed warm-up program purported to reduce knee injury had no effect on high risk biomechanics [39]. Cumulatively, there is evidence that demonstrates positive steps have been made toward prevention of ACL injuries in female athletes by using neuromuscular training [14]. However, current ACL reduction program designs that offer non-targeted training protocols may not be the most effective.

A linear regression analysis was performed on the current data to determine the potential to change high risk knee abduction moments with training. The regression analysis suggested that a majority of the potential to reduce this risk factor was related to their initial measure. The analysis indicated that athletes with what is described in the current manuscript as "low risk" will have little, if any, reduction of knee abduction torque from neuromuscular training. However, athletes who demonstrate increased knee abduction moments, and were classified as high risk, appear to have a greater potential to change this outcome measurement. This inference has been previously demonstrated [40], but is often ignored in clinical investigations [41]. The current model indicates that neuromuscular training targeted to female athletes with high knee abduction loads will most likely provide the greatest potential to significantly reduce dangerous knee loading profiles that increase their risk for ACL injury. A recent investigation by Grindstaff and colleagues indicated that standard, non-targeted neuromuscular training programs require application to 89 female athletes to prevent one ACL injury[42]. Theoretically, through identification of female athletes at greater risk for ACL injury, the numbers needed to treat to prevent an ACL injury could be substantially reduced. These investigations suggest that future interventional cohort investigations aimed to prevent ACL injury should apply their neuromuscular training intervention to high risk populations in order to improve the potential prophylactic effects. In addition, further research is warranted to delineate the most efficient training methods and the most effective means to increase widespread implementation of the most effective interventions.

## Limitations

The current study results may be limited by the selection of 25.25 Nm knee abduction to classify a female as "high-risk" for ACL injury. However, a value greater than 25 Nm would likely place the knee ligaments into the high load segment of the force length curve which may be magnified during high load activities [30]. In addition, the logistic regression that indicated maximal sensitivity and specificity for prediction of ACL injury risk using external knee abduction (25.25 Nm cutoff) was performed on a similar population to those trained in the present study (age, sports, height and weight) and tested the same DVJ activity as the current study [6]. Another potential limitation for the clinical application of the current study results is that determination of knee abduction load requires labor intensive testing methods that make it difficult to perform on a large scale. Current investigations are being conducted to help develop simpler testing techniques that can accurately and precisely identify female athletes who utilize increased knee abduction loads during sports related activities. Lastly, future investigations that include larger sample sizes of both test and control subjects will allow for the use of a more robust statistical analysis to further delineate the effects of neuromuscular training on high-risk populations.

### Clinical relevance

Considering the significant short and long-term debilitation associated with non-contact ACL injury, prevention of such injuries is crucial. The current paper addresses the potential to reduce ACL injury risk by examining the effects of well-established training strategies on lower limb mechanical parameters suggested to increase the risk of ACL injury. Specifically, we have taken steps to determine the most effective and efficient means of modifying potentially "high-risk" movement patterns in the female athlete. Though the "high-risk" movement and load patterns have been identified in the literature, their manifestation within precise injury mechanisms remains largely unclear. For example, how much knee abduction load is "too much", and furthermore, are injury causing load magnitudes largely subject specific? While the high-risk female athletes reduced their ACL injury risk factor of knee abduction in the current study, the 13% reduction did not take their mean score out of the designated high-risk category. Further research is needed to determine if greater volume, time of application (in-season vs. pre-season) or if potentially better techniques (e.g. biofeedback, education) are able to induce even greater reduction in high-risk knee mechanics and ACL injury risk in female athletes. Until such information is available, positive modifications in potentially "high-risk" movement strategies could be considered clinically significant, but not fully resolved in this population.

## Conclusion

A final step for intervention to a particular injury etiology is the appropriate application of a treatment to the population at risk. Current projects in our laboratory are aimed at the utilization of outlined neuromuscular screening techniques to identify potential "high-risk" female athletes. The goal should be to target identified "high-risk" females with the most appropriate neuromuscular training for their specific observed deficits. The current study indicates that female athletes categorized as high-risk based on previous coupled biomechanical and epidemiologic studies are more responsive to neuromuscular training [6]. However, the mean values were not reduced to values similar to the low-risk group. Thus, current exercise prescriptions of 4–7 weeks of isolated pre-season or in-season training may not provide the needed training dosage to reduce ACL injury risk in females [38,39,43]. Future work should aim to improve both efficacy and efficiency of neuromuscular training designed to target risk factors of ACL injury.

## Competing interests

The author(s) declare that they have no competing interests.

## Authors' contributions

GDM conceived the study, performed the biomechanical testing, carried out the neuromuscular training, participated in the data management and statistical analysis and drafted the manuscript. KRF participated in the study design, performed the biomechanical testing, conducted the biomechanical data analysis, participated in the data management and statistical analysis and helped to draft the manuscript. JLB performed the biomechanical testing, carried out the neuromuscular training, participated in the data management and helped to draft the manuscript. TEH participated in the study design and coordination and helped to draft the manuscript. All authors read and approved the final manuscript.

## Pre-publication history

The pre-publication history for this paper can be accessed here:


